# Association Between Korean-Style Balanced Diet and Risk of Abdominal Obesity in Korean Adults: An Analysis Using KNHANES-VI (2013–2016)

**DOI:** 10.3389/fnut.2021.772347

**Published:** 2022-01-20

**Authors:** Hye Jeong Yang, Min Jung Kim, Haeng Jeon Hur, Byoung Kook Lee, Myung-Sunny Kim, Sunmin Park

**Affiliations:** ^1^Department of Food Functionality Research, Korea Food Research Institute, Wanju-gun, South Korea; ^2^Department of Preventive Medicine, Soonchunhyang University, Asan, South Korea; ^3^Department of Food Biotechnology, Korea University of Science and Technology, Wanju-gun, South Korea; ^4^Department of Food and Nutrition, Obesity/Diabetes Research Center, Hoseo University, Asan, South Korea

**Keywords:** abdominal obesity, dietary patterns, waist circumferences, fermented foods, Korean health eating index

## Abstract

Abdominal obesity is a critical factor for metabolic diseases, and specific eating patterns such as the Mediterranean diet help prevent metabolic diseases. This study aimed to investigate the association between the modified Korean health eating index (MKHEI), including a Korean-balanced diet, and abdominal obesity risk according to genders in adults aged 20–64 years (4,886 males and 7,431 females), using the Korea National Health and Nutrition Examination Survey VI (2013–2016). Adjusted means and 95% confidence intervals of MKHEI scores and nutrient intake estimated using the 24-h recall method were calculated according to abdominal obesity (waist circumference ≥90 cm for men and ≥85 cm for women) after adjusting for age, residence area, region, education, income, drinking status, smoking status, marital status, and exercise. Adjusted odds ratios (ORs) for abdominal obesity were measured according to MKHEI tertiles using logistic regression analysis while controlling for covariates. Individuals aged >50 years, married, below high school, lower-income, heavy alcohol drinkers, past and current smokers, and males living in the southern areas had a higher risk of abdominal obesity. In both genders, the scores of all MKHEI components were lower in the abdominal obesity group (*n* = 2,895) than in the control group (*n* = 9,422). Further, the scores of fruits with and without fruit juice and those of beans, including fermented beans, were lower in the abdominal obesity group only in females but not in males. Further, the scores of fast foods were higher in the abdominal obesity group than in the control group only in females. After adjusting for covariates, the adjusted OR for abdominal obesity was inversely associated with Korean balanced diet (KBD) related to KHEI scores. Unlike KBD, MKHEI of Western-style diet was not associated with abdominal obesity in either gender. In conclusion, KBD can lower the risk of abdominal obesity in females and should thus be recommended to prevent abdominal obesity.

## Introduction

Notably, obesity is a worldwide epidemic affecting all age groups. Obesity is defined as excessive fat accumulation, but fat distribution affects metabolic disorders differently (1). Abdominal obesity is strongly associated with metabolic disease risk even in non-obese people, especially Asians (2). Ideally, abdominal obesity is determined by measuring fat in the abdomen using magnetic resonance imaging and computed tomography (3). However, these methods are expensive and difficult to perform. In clinical settings, abdominal obesity is estimated using waist circumference since it is significantly associated with intra-abdominal fat and is a reliable measure of abdominal obesity (3). Abdominal obesity is a primary risk factor for insulin resistance and systemic inflammation strongly associated with cardiovascular diseases, type 2 diabetes, Alzheimer's disease, and osteoarthritis (4). Furthermore, waist circumference is closely related to metabolic diseases and is a better indicator for metabolic disorders than body mass index (BMI) (5). The World Health Organization has established waist circumference cutoffs for abdominal obesity considering the development of metabolic diseases. The waist circumference cutoffs for abdominal obesity are lower in Asians than Caucasians (5).

Genetic and environmental factors affect abdominal obesity (6, 7). Environmental factors, including dietary patterns, exercise, smoking status, and alcohol intake, should be modulated despite personal genetic factors. However, the effects of environmental factors on abdominal obesity are complex and remain to be completely elucidated. Obesity such as abdominal obesity is determined by net energy intake and energy expenditure. The hypothalamus regulates energy balance to maintain normal body fat, particularly abdominal fat, by modulating hormones and neurotransmitters (7). However, measuring energy balance routinely can be challenging; thus, current strategies for preventing abdominal obesity do not involve counting the energy balance. Factors such as the timing of food intake, skipping breakfast, high energy food intake at dinner, and a midnight snack, are related to obesity regardless of energy balance (8). Carbohydrate and protein intakes are strongly associated with the timing of food intake for abdominal obesity (8).

Dietary patterns beneficially or adversely affect obesity irrespective of the energy balance (9). A Mediterranean diet is associated with preventive effects on metabolic syndrome, including obesity, regardless of the total energy intake (10). A Korean balanced diet (KBD) showed a similar effect on the Mediterranean diet in Korean adults; KBD reduces the risk of metabolic syndrome, whereas a Western-style diet (WSD) increases such risks (11). Asians have traditionally consumed grains as a staple food along with side dishes. However, the Korean diet has been westernized to include bread, noodles, and fast foods over the last several decades. KBD includes beans, fish, seaweeds, vegetables, fruits, and fermented foods, including kimchi, while WSD includes noodles, meats, soups, fast foods, and bread (11). Changes in the eating pattern may increase abdominal obesity risks.

Previous studies showed an inverse relationship between healthy eating habits determined by the healthy eating index (HEI) and abdominal obesity in various age groups and genders of several countries (12–14). Previous studies have some gender differences in eating habits on obesity and metabolic syndrome (15, 16). HEI is modified in different countries according to ethnic eating patterns (17–19). A Korean HEI (KHEI) reflecting healthy eating patterns has been developed by the Korea Centers for Disease Control and Prevention (17). KHEI demonstrates a part of KBD but cannot clearly distinguish between KBD and WSD intake. For example, the intake of meats, eggs, fish, and beans is categorized as one item in KHEI. The item needs to be divided into meats and eggs for WSD, fish for KBD, and beans and fermented beans for KBD in the present study. Moreover, KHEI did not include fermented vegetables, nuts, fast foods, and noodles to affect potentially health (20, 21). Therefore, KHEI has improved metabolic syndrome risk (16), but it cannot evaluate the KBD effect. Modified KHEI (MKHEI) reflecting KBD may explain the KBD effect on abdominal obesity. This study aimed to investigate the association between MKHEI scores and abdominal obesity risk in men and women. We hypothesized that the MKHEI, including items reflecting KBD and WSD, is associated with the risk of abdominal obesity determined by waist circumference according to gender. The hypothesis was assessed using Korea National Health and Nutrition Examination Survey (KNHANES)-VI (2013–2016) data.

## Methods

### Study Design and Population

This cross-sectional study analyzed the data of 12,317 adults (4,886 males and 7,431 females) aged 20–64 years from the KNHANES-VI 2013–2016. The participants were selected using a complex, stratified, multistage probability cluster survey to obtain a large representative sample of South Korean civilians (14). Health status data were collected by The Korean Centers for Disease Control and Prevention and the Korean Ministry of Health and Welfare. Health status was assessed using interviews, surveys, and physical examinations. The survey components and data collection methods have been detailed previously (22). The Institutional Review Board of the Korean Centers for Disease Control and Prevention (approval no. 2013-07CON-03-4C) approved the KNHANES, conducted according to the Helsinki Declaration of 1975, revised in 2008. The participants provided written informed consent to participate in this study.

### Data Collection and Variable Definitions

The health interview included age, gender, residence, education, income, alcohol intake, tobacco use, and regular exercise. Height and weight were measured with the participant wearing light clothing and no shoes. Obesity status was classified according to the recommendations of the International Obesity Task Force and the World Health Organization (WHO) Regional Office for Asians as follows: lean, BMI <18.5 kg/m^2^; normal, BMI ≥18.5– <25 kg/m^2^), and obese, BMI ≥25 kg/m^2^ (22). Area of residence was categorized into five regions: (1) Seoul, Incheon, Kyunggi area, and Gangwon-area; (2) Chunngchung area, Daejeon, and Sejong; (3) Kyungbook and Daegu; (4) Busan and Kyungnam area; and (5) Jeonbuk and Jeonnam areas, Kwangju, and Jeju areas (14, 17). Education level was categorized into < high school, high school, and ≥college. Family income was divided into four quartiles.

Usual alcohol intake was estimated by multiplying the average frequency (days per month) with the average amount of alcohol consumed on a single occasion determined in mL and by the type of alcoholic drinks ingested on a single occasion. Alcohol intake was converted into pure alcohol (in grams) consumed per day. Drinking habits were classified as none, mild (1–15 g), moderate (16–30 g), or heavy (>30 g) based on previous studies on the influence of alcohol consumption on alcohol-related diseases (23). Smokers were defined as smoking more than 100 cigarettes in their lifetime, while past smokers were specified as smokers who had not smoked for the last 6 months (23).

Regular exercise was defined as having ≥30 min of moderate exercise, including swimming slowly, playing doubles tennis or volleyball, and participating in occupational or recreational activities carrying light objects, at least 5 times per week or ≥20 min of vigorous exercise at least three times per week. The vigorous exercise included running, climbing, cycling fast, swimming fast, playing football, basketball, squash, singles tennis, jumping rope, and participating in occupational or recreational activities while carrying heavy objects (23). Marital status was categorized into married, divorced, widowed, and single.

### Abdominal Obesity Criteria

Abdominal obesity was defined using the cutoff of waist circumferences according to the World Health Organization guidelines for Asians and the Korean Obesity Society (24). The cutoff values for abdominal obesity were ≥90 cm in men and ≥85 cm in women (24). According to this criterion, the participants were divided into the abdominal obesity and control groups.

### Laboratory Tests

Serum was separated from blood samples collected after overnight fasting (at least 12 h). Serum glucose, high-density lipoprotein (HDL), triglyceride (TG), aspartate transaminase, and alanine transaminase concentrations were measured using an autoanalyzer (Hitachi Ltd., Tokyo, Japan). Serum low-density lipoprotein cholesterol (LDL) concentrations were estimated using the Friedwald equation as follows: LDL = total cholesterol – HDL – (TG/5), when TG was <400 mg/dL (15, 23). Serum TG concentrations were directly measured on a Hitachi 7,600 auto-analyzer in participants with ≥400 mg/dL. All biochemical assays were conducted at the Neodin Medical Institute, a laboratory certified by the Korean Ministry of Health and Welfare.

### Food and Nutrient Intakes From 24-h Recall and Food Frequency Questionnaire

Participants were asked about their usual food intake and dietary habits before the interview. Daily nutrient intake was estimated using a 24-h recall questionnaire administered by trained dieticians at mobile examination centers. Daily energy and nutrient intake were calculated using the Can-Pro 2.0, a nutrient assessment program developed by the Korean Nutrition Society. The nutrient intake from the 24-h recall was used for daily nutrient intake.

Usual food intake was determined using a semi-quantitative food frequency questionnaire (SQFFQ) designed and validated by the Ministry of Health and Welfare (18, 22). This SQFFQ questionnaire assessed the frequency and consumption amount of 113 food items during the previous year. Frequencies were checked using the following nine categories: never or seldom, once a month, 2–3 times a month, 1–2 times a week, 3–4 times a week, 5–6 times a week, once a day, twice a day, and three times or more every day (23). For foods consumed only once, the amount was categorized as half, one, or 1.5 of regular size.

### Modified Korean Healthy Eating Index (MKHEI) for Abdominal Obesity Risk

The KCDC designed and developed the KHEI to assess dietary quality in Koreans comprehensively. It includes the adequacy of food intake; appropriateness of saturated fat, sugar, and sodium intake; and balance of energy, fat, and carbohydrate intakes (25). KHEI scores were calculated using SQFFQ data. The KHEI is composed of adequacy (8 items), moderation (3 items), and balance (3 items) domains of energy intake. The scores of each item in adequacy part, moderation, and balance were described in a previous study (20). The KHEI scores were assigned using data from the Dietary Guidelines for Korean Adults and dietary reference intake for Koreans in 2015 (26).

We modified the KHEI to determine the effects of the primary components of KBD on abdominal obesity risk. MKHEI scores were assessed based on the SQFFQ data. The intakes of fermented vegetables, seaweed, fish, beans with fermented beans, and nuts were joined to MKHEI for the adequacy item. The moderation item also included fast food and noodle intake based on energy percentage. Vitamin C, fiber, and calcium intakes were added to the MKHEI for the balance item. The maximum scores for each item in the MKHEI are provided in Table 1. Each item was given a score from 0–5 or 0–10, with the total score ranging from 0 to 150. Higher scores in each item indicate healthier diets than lower scores. The standards of additional items were assigned based on the recommendations of the Food and Agriculture Organization and Korean dietary guidelines (25).

**Table 1:** The classification of each item in modified Korean health eating index.

**Components (score range)**	**Standard maximum score**	**Score**
Adequacy		
Have breakfast	5–7 times/w	10
	3–5 times/w	7
	1–3 times/w	4
	0 times	0
Mixed grains intake	≥0.8 serving/d	5
	0.1–0.8 serving/d	3
	<0.1	0
Fresh fruit intake	Men aged 19–64 years: ≥ 1.5 serving/d, Women aged 19–64 years: ≥ 1 serving/d; Men aged ≥ 65 years: ≥ 1 serving/d, Women aged ≥65 years: ≥ 0.5 serving/d	5
	Men aged 19–64 years: 0.5–1.5 serving/d, Women aged 19–64 years: 0.3–1 serving/d; Men aged ≥65 years: 0.3–1 serving/d, Women aged ≥65 years: 0.2–0.5 serving/d	3
	Men aged 19–64 years: 0–0.5 serving/d, Women aged 19–64 years: 0–0.3 serving/d; Men aged ≥65 years: 0.3–1 serving/d, Women aged ≥65 years: 0.2–0.5 serving/d	0
Vegetables intake excluding Kimchi and pickled vegetables intake	Men and women aged 19–64 years: ≥ 5 serving/d; Men aged ≥65 years: ≥ 5 serving/d, Women aged ≥65 years: ≥ 3 serving/d	5
	Men and women aged 19–64 years: 2–5 serving/d; Men aged ≥65 years: 2–5 serving/d, Women aged ≥65years: 2–3 serving/d	3
	Men and women aged 19–64 years: <2 serving/d; Men aged ≥65 years: <2 serving/d, Women aged ≥65 years: <2 serving/d	0
Fermented vegetables kimchi and pickled vegetable	≥3 serving/d	5
	1–3 serving/d	3
	<1 serving/d	0
Seaweed intake	≥2.5 serving/week	5
	0.5–2.4 serving/week	3
	<0.5 serving/week	0
Fish	Men aged 19–64 years: ≥ 2 serving/d, Women aged 19–64 years: ≥ 1.5 serving/d; Men aged ≥65 years: ≥ 1.5 serving/d, Women aged ≥65 years: ≥ 1 serving/d	5
	Men aged 19–64 years: 1–2 serving/d, Women aged 19–64 years: 1–2 serving/d; Men aged ≥65 years: 1–1.5 serving/d, Women aged ≥65 years: 0.5–1 serving/d	3
	Men aged 19–64 years: <1 serving/d, Women aged 19–64 years: <1 serving/d; Men aged ≥65 years: <1 serving/d, Women aged ≥65 years: <0.5 serving/d	0
Meat & eggs	Men aged 19–64 years: 2–3 serving/d, Women aged 19–64 years: 1.5–2 serving/d; Men aged ≥65 years: 1.5–2 serving/d, Women aged ≥65 years: 1.5–2 serving/d	5
	Men aged 19–64 years: <2 serving/d, Women aged 19–64 years: <1.5 serving/d; Men aged ≥65 years: <1.5 serving/d, Women aged ≥65 years: <1.5 serving/d	3
	Men aged 19–64 years: ≥4 serving/d, Women aged 19–64 years: ≥3 serving/d; Men aged 65 ≥years: ≥3 serving/d, Women aged ≥65 years: ≥ 2.5 serving/d	0
Beans including fermented beans	Men aged 19-64 years: ≥ 1 serving/d, Women aged 19-64 years: ≥ 1 serving/d; Men aged ≥65 years: ≥ 0.5 serving/d, Women aged ≥65 years: ≥ 0.5 serving/d	5
	Men aged 19–64 years: 0.5–1 serving/d, Women aged 19–64 years: 0.5–1 serving/d; Men aged ≥65 years: <0.5 serving/d, Women aged ≥65 years: <0.5 serving/d	3
	Men aged 19–64 years: <0.5 serving/d, Women aged 19–64 years: <0.5 serving/d; Men aged ≥65 years: <0.5 serving/d; Women aged ≥65 years: <0.5 serving/d	0
Milk and milk products intake	≥1 serving/d	10
	0.5–1 serving/d	5
	<0.5 serving/d	0
Nuts	≥2 serving/week	5
	0–2 serving/week	3
	0 serving/week	0
Moderation		
Percentage of energy from saturated fatty acids	≤ 7% of total energy intake	10
	7–9%	7
	9–11%	4
	>11%	0
Percentage of energy from polyunsaturated fatty acids	≤ 7% of total energy intake	4
	7–9%	10
	≥9%	7
Sodium intake	≤ 2,000 mg/d	10
	2,000–3,000 mg/d	7
	3,000–4,000 mg/d	4
	>4,000 mg/d	0
Percentage of energy from sweets and beverage	<10% of total energy intake	10
	10–15%	7
	15–20%	4
	≥20%	0
Noodle intake	≥4 serving/week	0
	0.5–4 serving/week	3
	<0.5 serving/week	5
Fast foods	≥4 serving/week	0
	0.5–4 serving/week	3
	<0.5 serving/week	5
Balance of nutrient intake	
Energy intake	75–120% of the estimated energy intake requirement (EER)	5
	65–75 or 120–135%	3
	<65 or >135%	0
V-C intake	Men aged 19–64 years: ≥100 mg/d, Women aged 19–64 years: ≥100 mg/d; Men aged ≥65 years: ≥100 mg/d, Women aged ≥65 years: ≥100 mg/d	5
	Men aged 19–64 years: 75–100 mg/d, Women aged 19–64 years: 75–100 mg/d; Men aged ≥65 years: 75–100 mg/d, Women aged ≥65 years: 75–100 mg/d	3
	Men aged 19–64 years: <75 mg/d, Women aged 19–64 years: <75 mg/d; Men aged ≥65 years: <75 mg/d, Women aged ≥65 years: <75 mg/d	0
Fiber intake	Men aged 19–64 years: ≥ 25 g/d, Women aged 19–64 years: ≥20 g/d; Men aged ≥65 years: ≥25 g/d, Women aged ≥65 years: ≥20 g/d	5
	Men aged 19–64 years: 15–25 g/d, Women aged 19–64 years: 10–20 g/d; Men aged ≥65 years: 15–25 g/d, Women aged ≥65 years: 10–20 g/d	3
	Men aged 19–64 years: <15 g/d, Women aged 19–64 years: <10 g/d; Men aged ≥65 years: <15 g/d, Women aged ≥65 years: <10 g/d	0
Ca intake	Men aged 19–64 years: ≥780 mg/d, Women aged 19–64 years: ≥730 mg/d; Men aged ≥65 years: ≥700 mg/d, Women aged ≥65 years: ≥800 mg/d	5
	Men aged 19–64 years: 630–780 mg/d, Women aged 19–64 years: 540–730 mg/d; Men aged ≥65 years: 570-700 mg/d, Women aged ≥65 years: 560-800 mg/d	3
	Men aged 19–64 years: <630 mg/d, Women aged 19–64 years: <540 mg/d; Men aged ≥65 years: <570 mg/d, Women aged ≥65 years: <560 mg/d	0
Percentage of energy from carbohydrate	55–65% of total energy intake	5
	50–55 or 65–70%	3
	<50 or >70%	0
Percentage of energy intake from fat	15–30% of total energy intake	5
	10–15% or 30–35%	3
	<10 or >35%	0

### Statistical Analysis

Descriptive statistics were evaluated using the frequency distributions of categorical demographic variables, such as obesity and lifestyle factors. Total KHEI scores were divided into three groups by tertiles, with the lowest tertile (T1) representing the worst dietary habit. Statistical differences were determined using chi-squared tests. Adjusted means and 95% CI of the MKHEI scores and mean intakes of major nutrients were analyzed using the Satterthwaite chi-square test according to gender. Finally, adjusted odds ratios (ORs) and 95% confidence intervals (CI) for the risk of abdominal obesity according to the MKHEI tertile scores were calculated using logistic regression after adjusting for age and residence area for model 1, covariates in model 1 plus occupation, income, education, and marital status for model 2, and covariates in the model 2 plus alcohol intake, smoking status, and physical activity for model 3. All statistical analyses were conducted using SAS version 9.4 (SAS Institute, Cary, NC, USA) or SUDAAN 11.0 (Research Triangle Institute, Research Triangle Park, NC, USA), which incorporates sample weights and adjusted for covariates in the statistical analysis of the survey design study. Statistical significance was set at *P* < 0.05.

## Results

### General Characteristics of the Study Population

There were significant differences in age, gender, education, income, smoking and alcohol intake, marital status, and survey years between the abdominal obesity and control groups (Table 2). The incidence of abdominal obesity was higher in men than women (*P* < 0.01), while it increased with age (*P* < 0.01). It was higher in participants living in rural areas than in the city. The participants with higher education and income were a much lower incidence of abdominal obesity (*P* < 0.01; Table 2). Current smokers had lower abdominal obesity incidence than non-smokers and past-smokers, while mild drinkers had lower incidence than the others (*P* < 0.01; Table 2). Married individuals had a higher incidence of abdominal obesity than unmarried ones. The rate of abdominal obesity was markedly higher in the 2015 and 2016 surveys than in the 2013 and 2014 surveys (Table 2).

**Table 2:** Distribution of study population by abdominal obesity according to socioeconomic and life style variables.

**Classification variables**		**Abdominal obesity**		
		**Yes (*N* = 2895)**	**No (*N* = 9422)**	*P* value^*^
Sex	Female	1,486 (18.5)	5,945 (81.5)	<0.01
	Male	1,409 (27.7)	3,477 (72.3)	
Age group	20–29	263 (15.5)	1,516 (84.5)	<0.01
	30–39	612 (22.6)	2,248 (77.4)	
	40–49	678 (23.2)	2,412 (76.8)	
	50–59	855 (26.5)	2,296 (73.5)	
	60–64	487 (32.5)	950 (67.5)	
Residence	Urban	2,288 (22.1)	7,957 (77.9)	0.02
	Rural	607 (28)	1,465 (72)	
Region	Region 1	1,044 (22.4)	3,599 (77.6)	<0.01
	Region 2	815 (21.4)	2,857 (78.6)	
	Region 3	325 (23.6)	973 (76.4)	
	Region 4	333 (24.7)	987 (75.3)	
	Region 5	378 (26.5)	1,006 (73.5)	
Education	< high school	728 (31.9)	1,466 (68.1)	<0.01
	High school	864 (23.9)	2,710 (76.1)	
	College	1,303 (20.2)	5,246 (79.8)	
Income	1st Q	361 (28.9)	745 (71.1)	<0.01
	2nd Q	748 (24.6)	2,223 (75.4)	
	3rd Q	926 (23.1)	2,997 (76.9)	
	4th Q	852 (20.3)	3,427 (79.7)	
Smoking status	Current smoker	1,581 (19.4)	6,148 (80.6)	<0.01
	Past smoker	589 (27.4)	1,467 (72.6)	
	Non-smoker	725 (28.3)	1,807 (71.7)	
Drinking status	None	770 (25.8)	2,217 (74.2)	<0.01
	Mild	1,349 (19.5)	5,269 (80.5)	
	Moderate	339 (24.6)	1,021 (75.4)	
	Heavy	437 (31.5)	915 (68.5)	
Exercise	Yes	1,439 (23.1)	4,621 (76.9)	0.79
	No	1,456 (22.8)	4,801 (77.2)	
Marriage	Yes	2,511 (24.8)	7,521 (75.2)	<0.01
	No	379 (17.2)	1,900 (82.8)	
Year	2013	643 (19.2)	2,618 (80.8)	<0.01
	2014	629 (20.6)	2,351 (79.4)	
	2015	792 (25.9)	2,159 (74.1)	
	2016	831 (26.2)	2,294 (73.8)	

*Values represented number of the participants and percentages*.

* *Chi square test for each classification variables for metabolic syndrome*.

*The cutoff values for abdominal obesity were ≥90 cm in men and ≥85 cm waist circumferences in women. CI, confidence intervals*.

*Region 1, Seoul, Incheon, Kyunggi area and Gangwon-area; Region 2, Chunngchung area, Daejeon, and Sejong; Region 3, Kyungbook, and Daegu; Region 4, Busan and Kyungnam area; Region 5, Jeonbuk and Jeonnam area, Kwangju, and Jeju area*.

### Daily Nutrient Intake From 24-h Recall

Daily energy intake was closely significantly higher in the abdominal obesity group than in the control group in men (*P* = 0.089) and women (*P* = 0.055). Fat, protein, and carbohydrate intakes based on daily energy intake (energy percent) did not differ between the abdominal obesity and control groups in both genders (Table 3). However, the carbohydrate intake tended to be lower in the abdominal obesity than the control (*P* = 0.051). The daily intake (g/day) of saturated fatty acid, monounsaturated fatty acid, and polyunsaturated fatty acid did not differ between the two groups in females. However, in males, polyunsaturated fatty acid intake was higher in the abdominal obesity group than in the control group (*P* = 0.014). Fiber intake was also higher in the abdominal obesity group than the control group in males, but not females (*P* = 0.036). The intakes of calcium, iron, and vitamin A were also not significantly different between the two groups in both genders (Table 3). Interestingly, sodium intake was lower in the abdominal obesity group than in the control group in men (*P* = 0.033) and women (*P* = 0.063), while daily vitamin C intake was significantly lower in the abdominal obesity group than in the control group in women, but not males (Table 3).

**Table 3:** Adjusted mean^¶^ and 95% CI of major nutrient intake according to genders and abdominal obesity calculated by waist circumference.

	**Female**			**Male**		
	**Abdominal obesity**	**Normal-weight**	*P* value^*^	**Abdominal obesity**	**Normal-weight**	*P* value^*^
Energy (Kcal/d)	1,845 (1,807~1,884)	1,803 (1,783~1,824)	0.055	2,443 (2,391~2,495)	2,390 (2,357~2,423)	0.089
CHO (En %)	65.07 (64.62~65.51)	65.18 (64.97~65.39)	0.644	61.01 (60.56~61.46)	61.54 (61.24~61.84)	0.051
Protein (En %)	13.47 (13.33~13.61)	13.53 (13.47~13.60)	0.396	12.63 (12.50~12.77)	12.65 (12.57~12.74)	0.787
Fat (En%)	18.36 (18.04~18.68)	18.57 (18.41~18.73)	0.249	17.71 (17.4~18.02)	17.50 (17.31~17.70)	0.273
SAF (g/d)	11.31 (10.92~11.69)	11.15 (10.96~11.35)	0.473	14.94 (14.43~15.46)	14.54 (14.23~14.84)	0.179
MUFA (g/d)	11.91 (11.52~12.30)	11.74 (11.53~11.96)	0.447	15.91 (15.34~16.48)	15.25 (14.91~15.59)	0.053
PUFA (g/d)	10.53 (10.19~10.86)	10.34 (10.16~10.52)	0.331	12.89 (12.45~13.33)	12.25 (11.98~12.52)	0.014
Fiber (g/d)	20.14 (19.61~20.67)	20.25 (19.96~20.53)	0.702	21.88 (21.23~22.53)	21.09 (20.70~21.48)	0.036
Ca (mg/d)	469.9 (456.4~483.4)	463.7 (457.0~470.4)	0.395	528.5 (513.5~543.5)	522.5 (513.0~532.0)	0.496
Fe (mg/d)	13.19 (12.87~13.50)	13.09 (12.91~13.26)	0.559	15.42 (15.01~15.82)	15.05 (14.79~15.31)	0.125
Na (mg/d)	3,171 (3,084~3,258)	3,083 (3,036~3,129)	0.063	3,912 (3,791~4,033)	3,762 (3,688~3,837)	0.039
Vitamin C (mg/d)	116.9 (112.3~121.5)	125.0 (122.2~127.8)	0.002	104.4 (99.6~109.2)	105.5 (102.4~108.6)	0.709
Vitamin A (RE/d)	622.8 (603.9~641.8)	622.5 (612.2~632.9)	0.976	661.3 (639.7~682.8)	643.5 (630.1~656.8)	0.157

*The cutoff values for abdominal obesity were ≥90 cm in men and ≥85 cm waist circumferences in women. CI, confidence intervals; CHO, carbohydrate; En%, energy percentage; SAF, saturated fatty acid; MUFA, monounsaturated fatty acid; PUFA, polyunsaturated fatty acid; Ca, calcium; Fe, iron; Na, natrium*.

¶ *adjusted by age, residence, region, education, income, drinking status, smoking status, marriage, and exercise*.

* *P-value S_waite Chi-Square*.

### MKHEI Scores Between Abdominal Obesity and Normal-Weight According to Genders

The MKHEI scores were compared between abdominal obesity and normal-weight (control) groups according to gender (Table 4). Total MKHEI scores of adequacy items were higher in the abdominal obesity group than the control in women but not in males (*P* = 0.001; Table 4). Among the components of adequacy, the scores of fruits with and without fruit juice and beans, including fermented beans, were much lower in the abdominal obesity group than in the control group in females after adjusting covariates including age, residence area, region, education, income, drinking status, smoking status, marriage, and exercise (Table 4). However, having breakfast, and intake of mixed grain, kimchi, seaweed, fish, meat and eggs, milk and milk products, and nuts among the adequacy class did not show significant differences between the abdominal obesity and control groups in both genders (Table 4).

**Table 4:** Adjusted^¶^ means and 95% CI of modified Korean health eating index (MKHEI) scores according to genders and abdominal obesity calculated by waist circumference.

**Classification**		**Female (*****n*** **=** **7431)**			**Male (*****n*** **=** **4886)**		
	**Abdominal obesity** **(*n* = 1,486)**	**Normal (*n* = 5,945)**	***P* value**	**Abdominal obesity** **(*n* = 1,409)**	**Normal (*n* = 3,477)**	*P* value^*^	
Adequacy	Have breakfast	6.914 (6.703~7.124)	7.039 (6.92~7.157)	0.28	6.579 (6.364~6.793)	6.768 (6.625~6.911)	0.124
	Mixed grains intake	4.088 (3.977~4.199)	4.136 (4.083~4.189)	0.426	3.808 (3.695~3.921)	3.777 (3.702~3.852)	0.635
	Fresh fruit intake	3.606 (3.499~3.714)	3.812 (3.766~3.859)	0.001	2.391 (2.273~2.509)	2.499 (2.429~2.569)	0.111
	Total fruit intake	3.504 (3.395~3.613)	3.707 (3.658~3.755)	0.001	2.145 (2.032~2.257)	2.223 (2.153~2.293)	0.234
	Vegetables intake excluding Kimchi and pickled vegetables	4.88 (4.849~4.911)	4.901 (4.887~4.915)	0.221	4.814 (4.772~4.855)	4.823 (4.796~4.85)	0.683
	Fermented vegetables intake	4.422 (4.35~4.494)	4.451 (4.415~4.486)	0.468	4.274 (4.188~4.359)	4.276 (4.224~4.328)	0.965
	Seaweed intake	1.5 (1.388~1.612)	1.503 (1.447~1.559)	0.955	0.862 (0.767~0.978)	0.768 (0.717~0.82)	0.204
	Fish intake	1.486 (1.363~1.61)	1.577 (1.517~1.637)	0.17	1.56 (1.449~1.671)	1.547 (1.469~1.624)	0.841
	Meat and eggs	2.642 (2.537~2.747)	2.685 (2.63~2.74)	0.454	2.948 (2.85~3.046)	2.94 (2.878~3.002)	0.9
	Beans including fermented beans	1.112 (0.996~1.229)	1.279 (1.225~1.332)	0.011	1.34 (1.225~1.455)	1.374 (1.3~1.448)	0.615
	Milk and milk products	3.523 (3.28~3.766)	3.715 (3.588~3.842)	0.158	3.175 (2.941~3.41)	3.431 (3.274~3.587)	0.079
	Nuts	0.402 (0.3~0.503)	0.415 (0.367~0.463)	0.816	0.433 (0.331~0.535)	0.462 (0.397~0.526)	0.636
	Total MKHEI for adequacy	37.98 (37.34~38.62)	39.09 (38.75~39.44)	0.001	34.22 (33.55~34.90)	34.72 (34.28~35.16)	0.196
Moderation	Percentage of energy from saturated fatty acids	9.423 (9.358~9.488)	9.401 (9.367~9.435)	0.527	9.477 (9.41~9.544)	9.449 (9.409~9.489)	0.488
	Percentage of energy from polyunsaturated fatty acids	4.724 (4.606~4.842)	4.684 (4.629~4.738)	0.536	4.361 (4.277~4.445)	4.314 (4.262~4.365)	0.351
	Sodium intake	5.083 (4.873~5.293)	5.3 (5.19~5.41)	0.059	3.771 (3.549~3.993)	3.874 (3.734~4.013)	0.445
	Percentage of energy from sweets and beverage	3.717 (3.59~3.845)	3.632 (3.569~3.695)	0.222	3.953 (3.833~4.073)	4.007 (3.937~4.078)	0.434
	Percentage of fast foods from energy	3.781 (3.716~3.846)	3.858 (3.828~3.889)	0.033	3.439 (3.356~3.522)	3.501 (3.457~3.546)	0.201
	Percentage of noodles from energy intake	3.422 (3.347~3.496)	3.414 (3.379~3.449)	0.848	3.308 (3.228~3.388)	3.303 (3.252~3.353)	0.913
	Total MKHEI for moderation	30.01 (29.67~30.36)	30.18 (30.02~30.35)	0.365	28.25 (27.89~28.61)	28.38 (28.17~28.60)	0.532
Balance	Energy intake	3.931 (3.82~4.043)	3.949 (3.896~4.001)	0.784	3.839 (3.731~3.947)	3.982 (3.908~4.055)	0.033
	Vitamin C intake	2.899 (2.769~3.029)	3.133 (3.061~3.205)	0.002	2.523 (2.386~2.66)	2.579 (2.489~2.668)	0.491
	Fiber intake	3.604 (3.517~3.691)	3.614 (3.569~3.659)	0.838	3.757 (3.665~3.85)	3.717 (3.664~3.771)	0.464
	Calcium intake	1.165 (1.062~1.267)	1.128 (1.074~1.182)	0.505	1.627 (1.508~1.745)	1.546 (1.47~1.621)	0.249
	Percentage of carbohydrate from energy intake	3.197 (3.091~3.303)	3.203 (3.151~3.255)	0.914	3.242 (3.132~3.353)	3.282 (3.214~3.349)	0.545
	Percentage of fat from energy intake	4.077 (3.984~4.17)	4.138 (4.099~4.177)	0.238	4.059 (3.977~4.141)	3.997 (3.945~4.048)	0.2
	Total MKHEI for balance	18.03 (17.67~18.39)	18.29 (18.10~18.48)	0.187	18.3 (17.93~18.67)	18.29 (18.05~18.52)	0.95
	Total MKHEI	86.02 (85.17~86.87)	87.57 (87.13~88.01)	0.001	80.77 (79.96~81.58)	81.39 (80.86~81.92)	0.184

¶ *adjusted by age, residence, region, education, income, drinking status, smoking status, marriage, and exercise*.

* *P-value S_waite Chi-Square. The cutoff values for abdominal obesity were ≥90 cm in men and ≥85 cm waist circumferences in women*.

*CI, confidence intervals; fermented vegetables: kimchi and pickled vegetables*.

Total scores of the moderation were not significantly different between the abdominal obesity and control groups in both genders (Table 4). The MKHEI scores of the moderation items, including saturated fatty acids, unsaturated fatty acids, sodium, and sweets and noodle intake, showed no significant between abdominal obesity and control groups in both genders (Table 4). However, the score of fast food intake was lower in the abdominal obesity group than in the control group in females (*P* = 0.033), and the sodium intake score was closely significantly lower in the abdominal obesity group in females (*P* = 0.059; Table 4).

The total scores of the balance category were not significantly different between the two groups (Table 4). Among the MKHEI scores of balance items, the vitamin C intake score was lower in the abdominal obesity group than in the control group only in females (*P* = 0.002), while in males, the energy intake score was lower in the abdominal group (*P* = 0.033). However, fiber and Ca intakes were not significantly different between the abdominal obesity and control groups in both genders (Table 4). Carbohydrate and fat intake (energy percentage) did not differ between the abdominal obesity and control groups in both genders.

The total MKHEI scores for adequacy, moderation, and balance items were significantly lower in the abdominal obese group than in the control group, only in women but not in men (*P* = 0.001; Table 4). It indicated that the women following modified Korean healthy eating patterns exhibited a lower abdominal obesity risk.

### Association Between Abdominal Obesity and MKHEI Scores Based on SQFFQ

**Figure 1:**
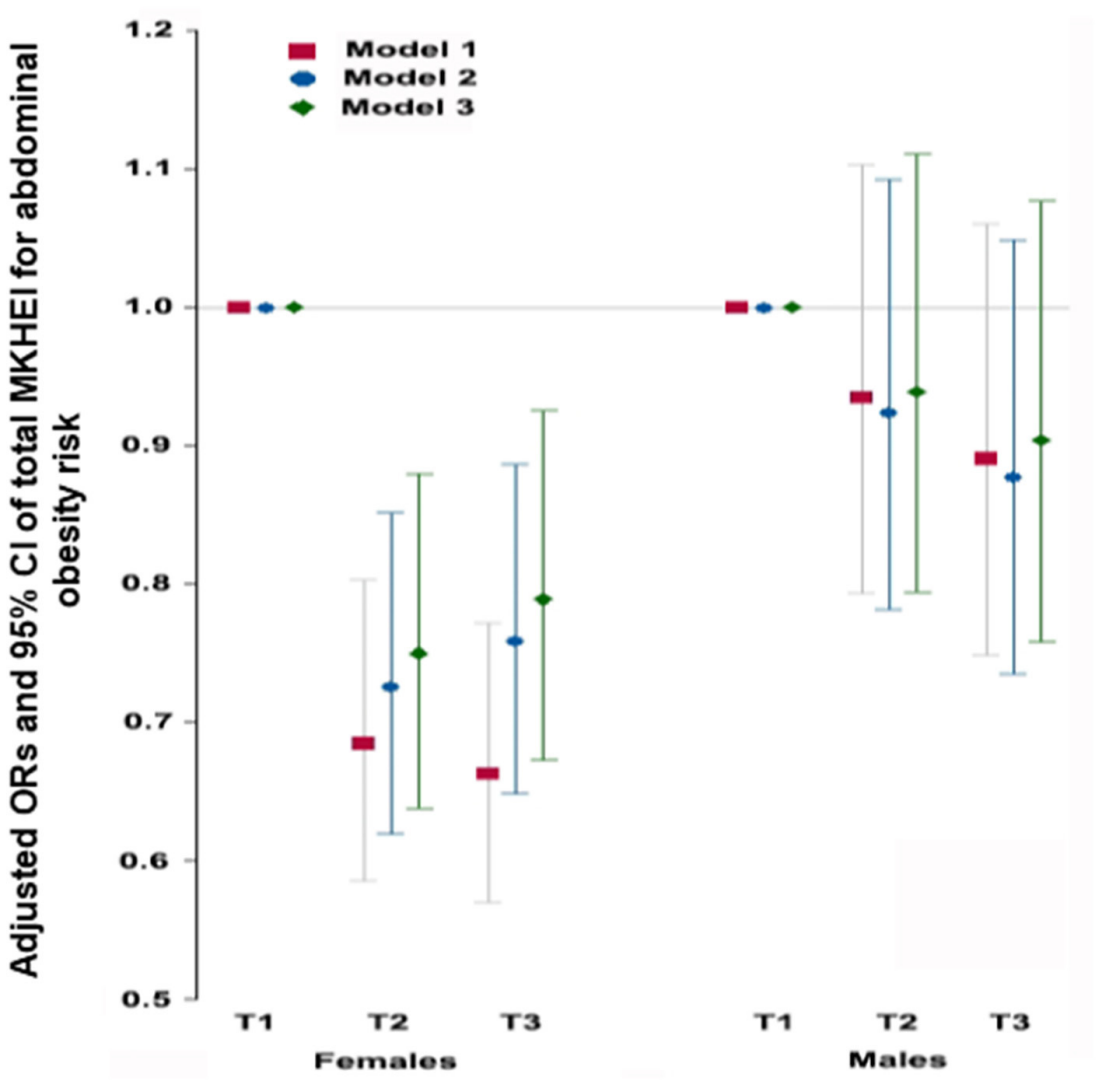
Adjusted odds ratios (95% CI) for abdominal obesity calculated by waist circumference after adjustments for covariates according to modified Korea Health Eating Index (KHEI). Model 1; adjusted for age and residence region. Model 2; adjusted for the covariates of model 1 + education, income, and marital status. Model 3: adjusted for the covariates of model 2+ smoking, alcohol, regular exercise.

The MKHEI scores were divided into tertiles, with T1 (the lowest score) as a reference. The covariates for model 1 included age and resistance region; those for model 2 contained covariates for model 1 plus education, income, and marital status; and those for model 3 were the covariates for model 2 plus smoking, alcohol, and regular exercise. In models 1, 2, and 3, the adjusted ORs of total MKHEI scores were inversely associated with abdominal obesity risk only in females (Figure 1). In model 3, the adjusted ORs and 95% CIs of T2 and T3 MKHEI for abdominal obesity were 0.789 (0.673–0.925) and 0.75 (0.638–0.88) in females after adjusting for covariates. It suggested that women with the highest intake (T3) of MKEHI were more likely to decrease by 25% to develop abdominal obesity risk. However, there were no significant differences in the adjusted ORs of MKHEI for abdominal obesity risk in males (Figure 1).

### Association of Abdominal Obesity With KBD- and WSD-Related MKHEI Scores

**Figure 2:**
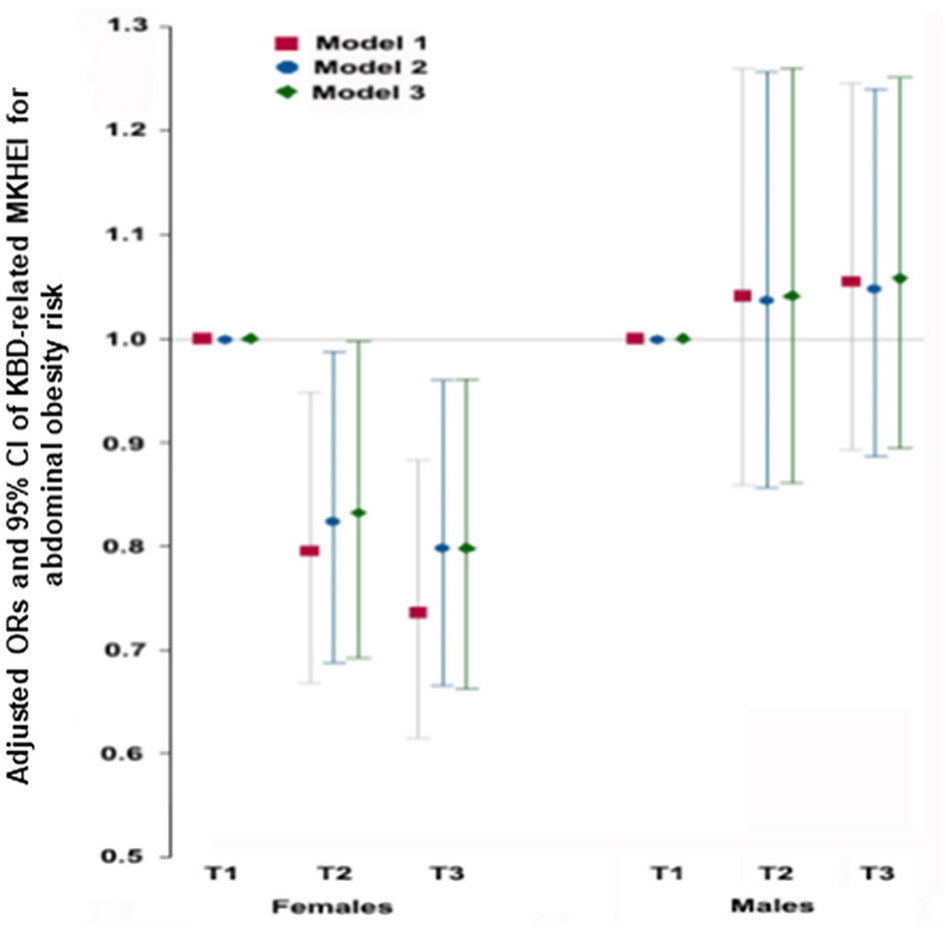
Adjusted odds ratios (95% CI) for obesity calculated by waist circumference after adjustments for covariates according to Korean balanced diet scores. Korean balanced diet scores were calculated by summing the mixed grains intake scores, vegetable intake excluding kimchi and pickled vegetable intake, fermented vegetables kimchi and pickled vegetables, fish, beans including fermented beans, seaweed intake, and percentage of energy from polyunsaturated fatty acids. Model 1; adjusted for age and residence region. Model 2; adjusted for the covariates of model 1 + education, income, and marital status. Model 3: adjusted for the covariates of model 2+ smoking, alcohol, regular exercise.

Among the MKHEI items based on the SQFFQ data, KBD was specifically related to seven items, namely, the intakes of mixed grains, vegetables excluding kimchi and pickled vegetables, fermented vegetables including kimchi and pickled vegetables, fish, beans including fermented beans, and seaweeds, and the percentage of polyunsaturated fatty acids from energy intake. The KBD-related items in MKHEI were added, and it was used as MKHEI for KBD. In all models, the adjusted ORs of the MKHEI scores for KBD were inversely associated with abdominal obesity risk in females. In model 3, the adjusted ORs and 95% CIs of the MKHEI for KBD were 0.832 (0.692–0.999) in T2 and 0.798 (0.662–0.961) in T3 for abdominal obesity risk after adjusting for age, residence region, education, income, marital status, BMI, smoking, alcohol consumption, and regular exercise, in females (Figure 2). It indicated that the adults with MKHEI for KBD were likely to decrease abdominal obesity risk by 20% in women. However, there was no association between MKHEI scores for KBD and abdominal obesity risk in males (Figure 2).

**Figure 3:**
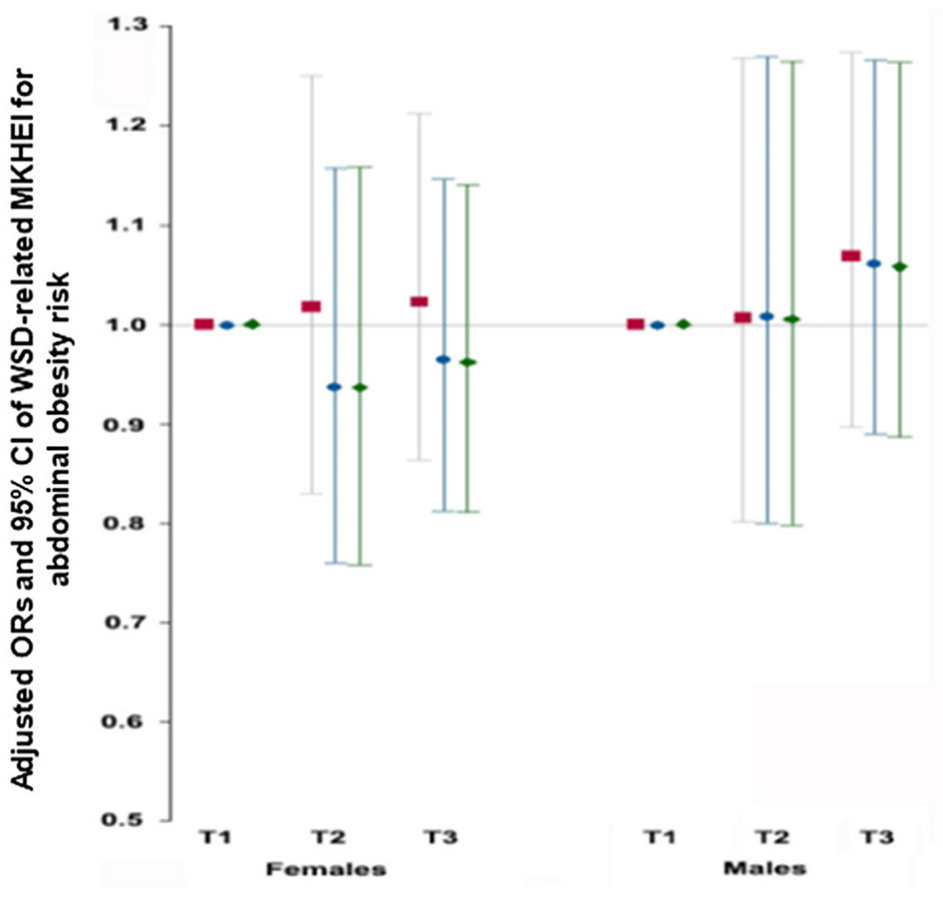
Adjusted odds ratios (95% CI) for obesity calculated by waist circumference after adjustments for covariates according to Western-style diet scores. Western-style diet scores were calculated by summing meat & eggs intake, noodle intake, percentage of energy from saturated fatty acids and fast foods. Model 1; adjusted for age and residence region. Model 2; adjusted for the covariates of model 1 + education, income, and marital status. Model 3: adjusted for the covariates of model 2+ smoking, alcohol, regular exercise.

MKHEI scores for WSD were calculated by summing the meat and egg, noodles, and fast food and the percentage of saturated fatty acids from energy intake. Unlike the MKHEI for KBD, WSD was not associated with abdominal obesity risk in models 1, 2, and 3 in both genders (Figure 3).

## Discussion

The present study showed that the total scores of the MKHEI and scores of KBD, but not those of WSD, were inversely associated with abdominal obesity only in women. Daily energy intake was tended to be higher in the abdominal obesity group than the control group in men (*P* = 0.089) and women (*P* = 0.055), although it was close to being estimated energy requirement (EER) of the participants. However, macronutrient intakes were not significantly different between the abdominal obesity and normal-weight control groups. Therefore, the adherence to Korean healthy eating patterns reflecting KBD has the potential to protect against abdominal obesity risk, especially in females. This study was the first study to show that a healthy eating pattern based on KBD can benefit abdominal obesity prevention in women.

Waist circumference as an indicator of abdominal obesity is associated with metabolic diseases. Although an energy imbalance mainly causes obesity, it is challenging to sustain balance for long periods. Not all obese persons consume higher energy intake than non-obese persons, and unhealthy eating habits are associated with abdominal obesity. In contrast, healthy eating habits are associated with health benefits, including preventing obesity, metabolic diseases, and certain cancers (10). The Mediterranean diet is recommended for a healthy eating pattern in Western countries to prevent obesity (10). However, it is challenging to apply the Mediterranean diet to the dietary patterns of Asians. Previous studies using KNHANES data have demonstrated that healthy eating patterns, including KBD, are inversely associated with the risk of metabolic syndrome and are positively associated with cardiovascular age (17, 27).

The prevalence of metabolic syndrome has increased from 2008 to 2017 in men but not in women (28). The prevalence of high waist circumference and serum triglyceride concentrations has also only increased in men. Further, although BMI changes are minimal (29), the proportion of participants with high blood pressure and fasting serum glucose concentrations increased from 2008 to 2017 in both genders (28, 29). These increases may be associated with lifestyle changes, including smoking, alcohol intake, and nutrient intake. Previous studies have demonstrated that alcohol intake and smoking are associated with metabolic syndrome risk (30). However, metabolic syndrome and its components have not been clarified to be related to nutrient intake and/or dietary patterns.

The Mediterranean diet and dietary approaches to stop hypertension (DASH) are reported to prevent abdominal obesity (31). Asians consume rice as a staple food and various foods as side dishes. Healthy diet patterns for Asians need to be specified, and thus, KEHI was modified to include items reflecting KBD, which is associated with a lower metabolic syndrome risk (32). KBD is composed of mixed grains with fish, seaweeds, beans, vegetables, fermented foods, and fruits, and it can be applied in Asians. In the present study, the total scores of the MKHEI were inversely related to abdominal obesity in both genders after adjusting for covariates. Moreover, the KBD-related MKHEI scores were inversely associated with abdominal obesity in women after adjusting for covariates, whereas there was no significant association between WSD and abdominal obesity. However, MKHEI in KBD was not related to BMI. Therefore, although higher KBD intake indicated a lower risk of abdominal obesity, KBD was not related to BMI in the present study.

Given that dietary patterns do not represent a person's total food intake unless evaluated using cluster analysis, the MKHEI for KBD included intakes of mixed grains, vegetables excluding kimchi and pickled vegetables, fermented vegetables kimchi and pickled vegetables, fish, beans including fermented beans, seaweeds and percentage of energy from polyunsaturated fatty acids. In the present study, MKHEI for KBD was inversely associated with abdominal obesity risk in females with energy intake similar to EER. Meanwhile, males had an energy intake of ~100 kcal/day above the EER, and the MKHEI for KBD was not significantly associated with abdominal obesity in males. Similarly, in cluster analysis of KBD participants with 11.6% higher energy intake based on EER, adults with KBD did not have significantly lower waist circumference and BMI than those with WSD and RMD (11). These results support that KBD is inversely associated with abdominal obesity and metabolic syndrome when people consume energy within the EER. The total energy intake and macronutrient intake did not influence abdominal obesity in the present study.

However, vitamin C intake was lower in the abdominal obesity group than in the control group in both genders, and these results were consistent with those in previous studies in different ethnicities and ages (33, 34). The difference in vitamin C is associated with a higher intake of fruit and fruit juice, including fruit juice, in both genders. Therefore, although ~10% of energy intake was higher than EER in the present study, abdominal obesity was mainly associated with fruit and vitamin C intake. Previous studies have consistently shown an inverse association between vitamin C intake, metabolic syndrome, and abdominal obesity (15, 35). Furthermore, serum vitamin C concentration is inversely associated with obesity and metabolic syndrome risk (36). Moreover, serum vitamin C concentrations are associated with obesity in both children and adults (36, 37). Lower vitamin C intake and serum vitamin C concentrations are associated with lower skeletal muscle mass in older males and females (38). In an animal study, vitamin C inhibited visceral obesity by activating peroxisome proliferator-activated receptor-α and increasing the mRNA expression of peroxisome proliferator-activated receptor (PPAR)-α-dependent fatty acid β-oxidation genes in high-fat diets (39). Thus, vitamin C may be associated with increased fatty acid oxidation. Therefore, vitamin C may play a critical role in preventing abdominal obesity and the risk of metabolic syndrome in males and females.

The advantage of the present study is that, similar to the Mediterranean diet, high MKHEI scores for KBD were shown to be inversely associated with abdominal obesity using a rolling sampling design that involves a complex, stratified, multistage probability cluster survey designed to represent the adult population. It supports that KBD should be recommended for preventing abdominal obesity in Asians. However, there are also some limitations to the present study. First, causal relations could not be determined as this was a cross-sectional study. Second, food intake scores in the MKHEI were obtained from the SQFFQ with 116 commonly consumed foods, and the intake of some foods may have been underestimated or overestimated. Third, nutrient intake estimated from a 24-h dietary recall might not reflect the usual intake because daily variation cannot be applied. Further studies are needed to determine the direct relationship between KBD and abdominal obesity in a randomized clinical trial or Mendelian randomization study.

## Conclusion

In men, daily energy intake was the only factor to influence abdominal obesity determined by waist circumference, but any MKHEI score was associated with abdominal obesity risk. However, in women, several MKHEI scores in addition to daily energy intake influence abdominal obesity. Among the individual items of MKHEI, scores for fruit intake, including fruit juice, beans, and fermented beans, vitamin C and Na intake are inversely associated with abdominal obesity only in women. Moreover, MKHEI scores for KBD were inversely associated with abdominal obesity only in females. These findings support that the adherence to Korean healthy eating patterns reflecting KBD, particularly adequate fruit and vitamin C intake, have the potential to protect against abdominal obesity risk in women.

## Data Availability Statement

The original contributions presented in the study are included in the article/supplementary material, further inquiries can be directed to the corresponding authors.

## Ethics Statement

The studies involving human participants were reviewed and approved by The Institutional Review Board of the Korean Centers for Disease Control and Prevention (approval no. 2013-07CON-03-4C). The patients/participants provided their written informed consent to participate in this study.

## Author Contributions

SP and HY: methodology and investigation. BL and HY: formal analysis. SP: formal analysis and writing–original draft preparation. MK and HH: validation. M-SK: conceptualization, funding acquisition, writing—review, editing, and supervision. All authors contributed to the article and approved the submitted version.

## Funding

This research was supported by the Research Program of the Korea Food Research Institute (KFRI), funded by the Ministry of Science and ICT.

## Conflict of Interest

The authors declare that the research was conducted in the absence of any commercial or financial relationships that could be construed as a potential conflict of interest.

## Publisher's Note

All claims expressed in this article are solely those of the authors and do not necessarily represent those of their affiliated organizations, or those of the publisher, the editors and the reviewers. Any product that may be evaluated in this article, or claim that may be made by its manufacturer, is not guaranteed or endorsed by the publisher.
